# The Time Course of Attention: Selection Is Transient

**DOI:** 10.1371/journal.pone.0027661

**Published:** 2011-11-18

**Authors:** Anna Wilschut, Jan Theeuwes, Christian N. L. Olivers

**Affiliations:** VU University, Amsterdam, The Netherlands; Kyushu University, Japan

## Abstract

The time course of attention has often been investigated using a spatial cuing task. However, attention likely consists of multiple components, such as selectivity (resolving competition) and orienting (spatial shifting). Here we sought to investigate the time course of the selective aspect of attention, using a cuing task that did not require spatial shifting. In several experiments, targets were always presented at central fixation, and were preceded by a cue at different cue-target intervals. The selection component of attention was investigated by manipulating the presence of distractors. Regardless of the presence of distractors, an initial rapid performance enhancement was found that reached its maximum at around 100 ms post cue onset. Subsequently, when the target was the only item in the display, performance was sustained, but when the target was accompanied by irrelevant distractor items, performance declined. This temporal pattern matches closely with the transient attention response that has been found in spatial cuing studies, and shows that the selectivity aspect of attention is transient.

## Introduction

Attention is the set of mechanisms that the brain uses to control incoming information, by selecting physically salient or behaviourally meaningful parts for further processing. Important for understanding how selection takes place is to know its time course. When does attention enhance relevant information, and how does this enhancement develop over time?

Studies using variations of the spatial cuing task [1] have provided evidence for the existence of a transient component of the attentional response. This temporal pattern of attention was described in detail by Nakayama and Mackeben [2]. In their study participants were asked to identify a peripheral target bar in a visual search display that was filled with distractor bars, varying in luminance and orientation. The display was followed by a mask. Prior to the target, a peripheral cue indicated its location at varying cue-target stimulus onset asynchronies (SOA). The observers' task was first to tell whether the target was present, and if so, which one of two possible combinations of luminance and orientation it carried. When SOA was increased from 0 to 100 ms, target discrimination accuracy rose rapidly to its maximum, revealing a cue-induced enhancement of attention. This attentional enhancement was found to be transient, as accuracy declined when the SOA was further increased to several hundreds of milliseconds. This transient attention pattern has also been found by others in studies of spatial attention [2], [3], [4], [5], [6], [7], [8].

However, attention is likely not a unitary mechanism, but consists of multiple processes, each of which may have a different temporal profile. For example, based largely on spatial cuing, attention has been divided into three components [9], [10], [11], [12]. The nomenclature and scope of these have varied, but most conceptualizations include elements of spatial orienting, selectivity, and non-specific temporal or “warning” effects. Spatial orienting component has been postulated to enable the disengagement of attention from its current location and its shifting to a salient or relevant location [13], [14]. The selective component of attention has been linked to conflict resolution between relevant and competing irrelevant information. For example, items surrounding a target can cause competition by sensory crowding [15], [16], but also by interfering with the target representation at a higher level [17]. Selective attention may resolve this competition by enhancing the target processing or by suppressing the distractor processing [18], [19], [20]. The temporal component of attention has been linked to alerting, arousal, and foreperiod effects that occur when a warning signal is presented prior to a target. Performance is found to gradually improve when the SOA between warning signal and target is increased, which is thought to reflect increased perceptual sensitivity or preparedness to respond [10], [21], [22], [23]. This raises the question as to which element of attention is transient. The aim of the present study was to investigate the time course of attention in more detail, by focusing on the selectivity aspect of it, in the absence of a need for a spatial shift, and while controlling for general warning effects.

The time course of attentional selection in the absence of spatial shifting has been studied earlier by Weichselgartner and Sperling [24]. Their procedure used a rapid serial visual presentation (RSVP) task, in which a series of digits was presented in rapid succession (at about 10 items per second), all at the same location. One digit was assigned the role of first target, by cuing it with for example an outline square. The task was to report the target, but also the three digits following it. The likelihood of any item being reported suggested a fast and transient automatic component of attention – similar to that subsequently observed by Nakayama and Mackeben (1989) – as performance first rose steeply after the cue, but then rapidly declined again. However, the procedure used by Weichselgartner and Sperling may not have captured the time course of attentional selectivity as such, but possibly included other confounding factors. First, the task of Weichselgartner and Sperling, requiring the report of a series of digits after each cue, is likely to include a substantial memory component. The decline in reporting items at later time points after the cue might have indicated a limit in working memory capacity rather than a true ability to allocate visual attention. Second, their specific RSVP procedure meant that the cue was a) presented together with a target that needed to be reported, b) was followed by items that up till then were defined as distractors, and c) probably induced a task switch from having to ignore digits to having to report digits. Each of these factors could have contributed to the rapid decline of performance after the cue, for example by causing an attentional blink and/or a switch cost [25], [26], [27]. Thus, the question remains which component of attention is transient.

In order to investigate the time course of the selectivity aspect of attention we modified the task of Nakayama and Mackeben [2]. Two important alterations were made: First, we rendered spatial shifting unnecessary by presenting the cue and the target invariably at central fixation. Note that the study of Nakayama and Mackeben (1989) included an experiment in which the target was consistently presented at the same location. However, this was a location at the periphery while observers were required to always maintain fixation at the centre of the display. Spatial shifts of attention can therefore not be excluded. In another experiment, they used displays that were small enough to fit within the foveal region (i.e. within 1 degree of visual angle). However, here the target location was made uncertain again, thus potentially invoking the need for spatial shifts, even if only small.

Second, we manipulated selectivity by presenting the target either alone, or surrounded by distractors (a factor that was not included in the study of Nakayama and Mackeben). Presenting the target with competing proximal stimuli was assumed to reveal the effects of attentional selectivity, as has been suggested by the biased competition model [18] and the ambiguity resolution theory [19]. There is also direct evidence showing that the presence or absence of distractors affects the occurrence of cuing effects [28], [29]. In these studies, cuing benefits on target identification were found to be only present, or larger, when distractors accompanied the targets. However, because only one cue-target SOA of approximately 100 ms was used in these studies, it could not be investigated whether these effects were transient or not. Therefore, in the present study the effects of distractors on cuing at a single central location were studied across a number of SOAs. If the transient character of attention is tied to the selection of targets from competing objects, we should see this component emerge in conditions where distractors are present, but not where the distractors are absent.

Note that although the cue was hypothesized to aid selection of the target and shield it from competing objects, it may also serve as a general warning signal which alerts observers to the forthcoming target display, and hence improves overall performance. These effects have been found to be relatively slow however, with a steady improvement up to 500 to 1000 ms, whereas here we were interested in earlier, faster modulations of selective attention, akin to transient attentional enhancement. Furthermore, warning effects have been observed mostly as changes in reaction times, suggesting a locus at response preparation and response selection, with less clear implication on perception [21], [23], [but see 30], [31]. In any case, in our design performance in the distractors present condition was compared to that in distractors absent condition. A general temporal pattern as caused by alerting should be similar for both conditions, while a difference in selectivity should become obvious as an interaction between time and distractor presence.

## Experiment 1

**Figure 1:**
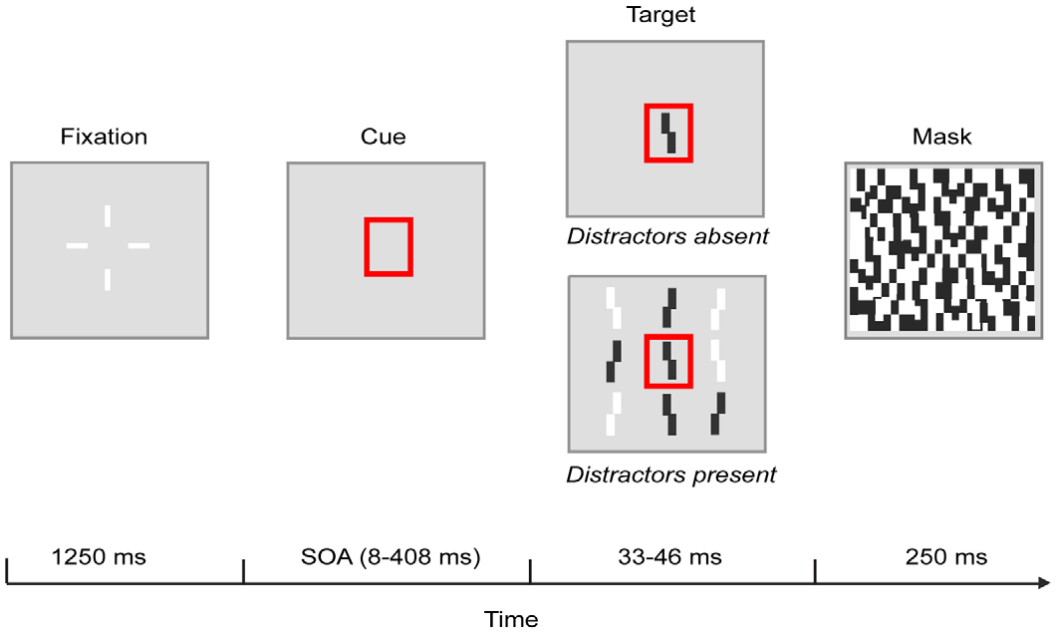
An outline of the task used in all experiments. The proportions are not drawn to scale. After the fixation cross, the cue was shown for a varying SOA (8–408 ms, depending on the experiment). The target was then shown with the cue (target duration being 16, 33, 42, or 46 ms, depending on the experiment), either alone (Distractors absent) or with surrounding distractors (Distractors present). The number of distractors was 20 in Experiments 1 and 3, and eight in Experiment 2 (in Experiment 4 there were no distractors). In Experiment 3 either a local or global cue was shown. The target and the distractors were eventually substituted by a mask.

In Experiment 1 we investigated the time course of attentional selection by looking at how a cue affects target processing with and without distractors, as a function of time. Our task (see Figure 1) was adopted from the one used by Nakayama and Mackeben [2], with the difference that the cue and the target were invariably shown at fixation. A red outline rectangle cue preceded a target item with five SOAs (8–408 ms). The target was a black or white vertical bar with a vernier acuity offset towards the left or right. A mask substituted the target after a short duration. Participants performed a discrimination task on the polarity as well as vernier offset direction. Selectivity was studied between two distractor conditions. In the Distractors absent condition targets were presented alone, whereas in the Distractors present condition the target was surrounded by distractors.

### 1.1. Methods

#### Participants

Ten students participated in Experiment 1 (five males, aged 19–23 years, mean 19.9 years), and received either course credits or money (7 €/hour). All reported normal or corrected-to-normal vision. Participants of this and the following experiments were drawn from the subject pool of the Faculty of Psychology and Education of the VU University. As part of the undergraduate participation scheme, they received written information about their rights as a participant in scientific studies. For each specific experiment, they then first received written on-line information about the nature of the experiment, before they consented to participation by signing up to the study. This was followed by verbal explanation and consent at the start of the experiment. The procedure was conducted in accordance with the Declaration of Helsinki, and ethical approval for this study was obtained from the Scientific and Ethical Board of the Faculty of Psychology and Education of the VU University (VCWE).

#### Stimuli and apparatus

The experiment took place in a dimly lit room. Stimulus presentation and data recording were carried out by an HP Compaq d530 CMT Pentium 4 computer with E-Prime software (Psychology Software Tools Inc.). The stimuli were viewed from about 70 cm distance on a 19-inch CRT monitor (1024×768 resolution, refreshing at 120 Hz). The background was grey (CIE: x = .295, y = .346; 36 cd/m^2^). All the stimuli were centred in the middle of the screen. A fixation cross was made of four orthogonal white (CIE: x = .295, y = .347; 95.8 cd/m^2^) line segments that pointed to the centre but left the target area at fixation empty. A red outline rectangle (CIE: x = .620, y = .344; 17.2 cd/m^2^) extending to 1.1° horizontally and 2.1° vertically served as a cue. Targets were 1.2° tall and 0.2° wide, black (CIE: x = .295, y = .344; 18 cd/m^2^) or white (CIE: x = .293; y = .346; 55 cd/m^2^) vertical bars. The upper half of the target bar had a 0.06° vernier offset shift to the left or to the right. In Distractors present condition, the target display also contained 20 irrelevant distractors that with the central target formed a 3×7 rectangular grid. The centre to centre distance between bars was 0.9° in horizontal and 2.1° in vertical direction. The distractors were vertical bars similar to the target, whose colour and offset direction were randomized over the 20 peripheral locations, with the restriction that half of the distractors were black and half of them were white. Similarly, the offset shift was to the left for half of the distractors, and to the right for the other half of the distractors. The mask was a 6.2°×6.2° square completely filled with scrambled black and white vernier offset bars that covered the entire area where the distractors could appear.

#### Design and procedure

Each trial began with a fixation display that was present for 1250 ms. Factors Cue presence, Distractor presence, and SOA were randomly varied within blocks. In the Cue present condition, the fixation cross was subsequently replaced by a cue that surrounded the central location for a variable duration. Five levels of SOA (8, 58, 108, 208, and 408 ms) were used. After the SOA the target appeared. In the Distractors absent condition the target was presented alone for 33 ms. In the Distractors present condition the target was accompanied by 20 distractors. Based on piloting studies showing that distractors made the task overall more difficult (as would be expected), the target duration was increased to 42 ms in this condition. In both groups, the cue remained on during the presentation of the target. The target display was followed by the mask for 250 ms. After the mask participants gave their response, followed by a 750 ms intertrial interval before the beginning of the next trial. The Cue absent condition was similar to the Cue present condition, except that instead of the cue, the fixation cross was on for an additional 200 ms, and thus only one ‘SOA’ was used. Immediately following the offset of the fixation, an uncued target was shown either alone (Cue absent/Distractors absent) or surrounded by distractors (Cue absent/Distractors present).

The task was to indicate by a key press whether the target bar was black or white and whether the vernier offset was to the left or to the right (four-alternative forced choice task). Participants were instructed to keep their eyes fixated in the centre of the screen during the whole experiment and respond as fast and accurately as possible, with the emphasis on accuracy. A brief high tone was played as feedback for a correct response. All participants began with a practice block of 144 trials. At the start of the practice the target presentation time was set to 183 ms from which it was gradually reduced every ten trials, provided that accuracy was above 75%. All except one of the participants improved during the practice so that they reached the 42 ms target duration. As a memory aid, throughout the practice trials only, the four different target bar types were presented at the foot of the display in the order that corresponded with the arrangement of the response keys on the keyboard (‘z’, ’x’, ‘n’, and ‘m’). After the practice, eight experimental blocks of 144 trials each were completed. This resulted in 96 trials for each condition (2 distractor conditions ×5 SOAs for the Cue present condition, and 2 distractor conditions for the Cue absent condition). Only the responses from the experimental blocks were used for the analyses. The experiment took about 70 minutes, and there were self-paced breaks between the blocks.

### 1.2. Results and Discussion

**Figure 2:**
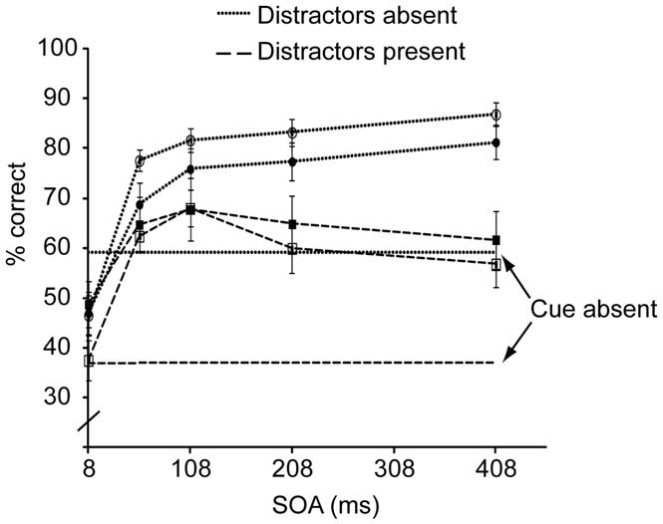
Target identification accuracy for Experiment 1 (filled symbols) and 1b (open symbols), plotted as a function of SOA, cue and distractor presence. The error bars represent the standard error of the mean.

Trials with excessively short (<200 ms) or long (>5000 ms) responses were discarded, resulting in exclusion of 0.7% of trials. For statistical tests an alpha level of .05 was used, adjusted by Bonferroni correction for t-test comparisons. If necessary, p-values were adjusted for sphericity violations by Greenhouse-Geisser corrected degrees of freedom. The accuracy scores for Experiment 1 are shown in Figure 2. For the Cue present condition, repeated measures ANOVAs with Distractor presence and SOA as within-subject variables revealed a significant main effect of Distractor presence on overall accuracy (F (1, 9) = 14.32, p<.01), with higher accuracy in the Distractors absent condition compared to the Distractors present condition (*M* = .70, *SD* = .17 vs. *M* = .62, *SD* = .18). Also the main effect of SOA was significant (F (4, 36) = 68.60, p<.001), as well as the interaction between Distractor presence and SOA (F (4, 36) = 14.30, p<.001). Accuracies rose rapidly with increasing SOA for both Distractors absent and Distractors present conditions up to 108 ms (8 vs. 108 ms SOA: t (9) = 11.15, p<.001 and t (9) = 7.74, p<.001, respectively). At later SOAs Distractors absent condition showed an even further improvement (108 vs. 408 ms: t (9) = 3.43, p<.01), whereas in the Distractors present condition the accuracy decreased (108 vs. 408 ms: t (9) = 3.57, p<.01).

Accuracy was significantly higher in the Cue present condition than in the Cue absent condition for all SOAs (all ps<.01; Cue absent/Distractors absent: *M* = .59, *SD* = .20; Cue absent/Distractors present: *M* = .37, *SD* = .13), except in the Distractors absent condition at 8 ms SOA, where accuracies were better when the cue was absent than when the cue was present (t (9) = 3.61, p<.01). One explanation for this could be that the cue simultaneously induced a partial masking effect at very short SOAs. The degrading effects of masking would then be overruled by the cued enhancement, except at the 8 ms SOA, when masking is expected to be strongest and cuing effects weakest. Overall, the results from the cue absent conditions serve to show that the cue indeed had a facilitating effect on target identification.

We also analyzed the RTs, although we emphasize that these were only secondary to the main dependent measure of accuracy (given a very brief and masked target presentations). Table 1 shows the RTs for Experiment 1 and the subsequent experiments. These were marginally affected by the Distractor presence (F (1, 9) = 4.09, p = .074). The effect of SOA was significant (F (4, 36) = 22.38, p<.001), whereas there was no interaction between Distractor presence and SOA (F (4, 36) = 1.76, ns.). The overall pattern followed that of the accuracy scores, with a more transient speeding of RTs in the distractor present condition. Although this did not bear out statistically, the RT pattern serves to demonstrate that no speed-accuracy trade-off underlies the results of interest (the accuracies).

**Table 1:** RTs for Experiments 1–[Sec s3] [Sec s4] 4.

	Distractors/Cue/Target duration (ms)	SOA (ms)									
		8	33	58	83	108	133	158	208	283	408
*Exp 1*	*Absent*	799±66		726±54		683±47			696±53		688±49
	*Present*	804±62		727±64		716±62			717±57		740±58
*Exp 1b*	*Absent*	790±42		696±32		681±26			662±26		664±25
	*Present*	820±43		739±34		706±31			708±31		729±27
*Exp 2*	*Absent*	838±35	776±31	747±17	722±19	717±31	706±24	711±22	694±16	698±15	719±20
	*Present*	845±60	800±60	767±56	756±54	758±63	755±62	748±55	761±59	781±62	806±62
*Exp 3*	*Local cue*	873±84		836±78		805±81			843±73		855±72
	*Global cue*	928±58		935±68		915±69			923±71		987±58
*Exp 4*	*16*			777±37		767±47			767±37		816±40
	*33*			754±40		733±41			699±36		788±59

Mean RTs and corresponding standard errors are shown by Condition (Exp 1 and 2: Distractors absent/present; Exp 3: Local/Global cue; Exp 4: Target duration 16/33 ms) and SOA.

The results of the cue present condition show that the identification of a target following a cue first rapidly improves up to 108 ms. After that, performance depends on the presence of irrelevant distractor objects. If the target is accompanied by distractors, performance is transient: It gradually declines when the SOA is increased to 408 ms. In contrast, if the target is presented alone, performance shows even further improvement. It seems that the cue initially enhances perception in a rather nonspecific manner, irrespective of whether the target is presented with distractors or not. This enhancement turns out to be transient however, but only under conditions when there is competition from multiple items. We suggest that it is this competition, or selectivity aspect, that is only transiently biased in favour of the target. When no such competition is present, as in the distractors absent condition, the cue equally enhances performance, but no decline occurs as there is no need for selection.

Note that the Distractor conditions differed not only by the presence of distractors, but also by the target duration, which was adjusted in order to reduce differences in overall difficulty. To be sure that this difference did not have an influence on the observed result pattern, in Experiment 1b we replicated Experiment 1, but now with the target duration made equal across conditions (always 33 ms). Experiment 1b was further identical to Experiment 1, except that there was no cue absent condition. Ten new observers participated (one male, aged 19–26 years, mean 21.3 years). The results, shown in Figure 2, replicate those of Experiment 1. Distractors absent as well as distractors present conditions show an initial rapid enhancement, while only the distractors present condition shows a decline. The effects of distractor presence and SOA were significant (F (1, 9) = 66.12, p<.001; F (2, 17.7) = 48.20, p<.001, respectively), as was their interaction (F (4, 36) = 14.31, p<.001). Again, the RT results (see Table 1) followed this pattern, as shown by the significant main effects (Distractor presence: F (1, 9) = 20.13, p<.01; SOA: F (1.82, 16.37) = 18.86, p<.001), and the interaction effect that now approached significance (F (4, 36) = 2.38, p = .07). Also here the responses were transiently speeded in the distractors present condition.

## Experiment 2

In Experiment 1 the distractor conditions were mixed. This means that on half the trials, no distractors were present, which may have reduced the effort that observers would put in using the cue, or in sustaining the selectivity. It is possible that observers may overcome the late selection deficit if they expect distractors to be present on every trial. We therefore conducted Experiment 2, in which distractor presence was manipulated between subjects, such that one group would always be faced with displays that contained distractors, while others were presented with targets alone. As a benefit, running the condition between subjects enabled us to include more SOAs per subject, and thus to investigate the actual peak of enhancement in finer detail.

### 2.1. Methods

Methods of Experiment 2 were largely similar to Experiment 1, with the following exceptions. Twenty students participated and were divided in two groups (Distractors absent group: N = 10, five males, aged 18–25 years, mean 21.6 years; Distractors present group: N = 10, two males, aged 19–25 years, mean 20.4 years). The cue was slightly enlarged to a square of 2.5°×2.5°. Additionally, eight distractors were presented in a square formation 2.1° around the target (centre to centre distance). Distractors were thus presented more sparsely and the separation between the cue and the target was larger, but the spatial spread of the task items was the same as in Experiment 1, covered completely by the mask. The levels of SOA was increased to ten (8, 33, 58, 83, 108, 133, 158, 208, 283, and 408 ms). For the Distractors absent group, the target was presented alone for 33 ms. For the Distractors present group the target duration was on average 46 ms (due to a minor technical problem, it was 42 ms on half of the trials and 50 ms on the other half, not confounded with the SOA condition). The experiment took about one hour.

### 2.2. Results and Discussion

**Figure 3:**
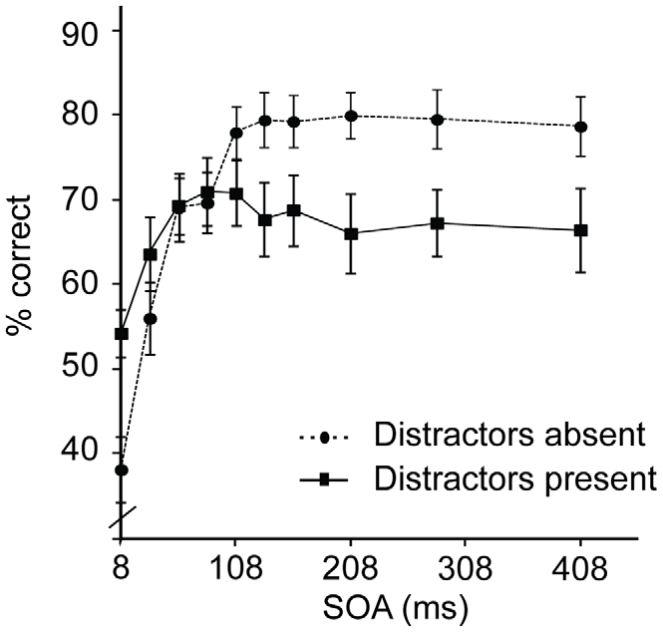
Target identification accuracy for Experiment 2, plotted as a function of SOA and distractor presence.

Discarding excessively short (<200 ms) or long (>5000 ms) responses resulted in exclusion of 0.2% of trials in the Distractors absent group and 0.7% in the Distractors present group. Accuracies for the two groups are presented in Figure 3. A two-way mixed ANOVA was performed with group as a between-subjects factor (two levels) and SOA (10 levels) as a within-subjects factor. Overall accuracy levels did not differ between the groups (F (1, 18)<1, ns.). However, the effect of SOA was highly significant (F (4.8, 86.9) = 48.17, p<.001), as well as the interaction between group and SOA (F (4.8, 86.9) = 15.33, p<.001). As can be seen from Figure 3, performance followed a different time course depending on whether distractors were absent or present. On the basis of Experiment 1, we planned the same comparisons, now one-tailed. Accuracy at 8 ms SOA started significantly lower in the Distractors absent group than in the Distractors present group (t (18) = 2.98, p<.01), but then rose rapidly up to about 108 ms (t (9) = 17.51, p<.001, for SOAs 8 vs. 108 ms). Afterwards it was sustained at a relatively constant level (t (9) = 0.39, ns, for SOAs 108 vs. 408 ms; although, numerically, the highest performance was reached at 208 ms). In the Distractors present group, accuracy rose for the first 83 ms (8 vs. 83 ms: t (9) = 5.49, p<.001). In contrast to the Distractors absent group, performance showed a decline at later SOAs (108 vs. 408 ms: t (9) = 2.09, p = .033). This narrowly failed to reach significance at Bonferroni corrected alpha levels, but since this serves as a replication of Experiment 1 (which was already replicated in Experiment 1b), we take this as evidence for a decline. Moreover, the decline was also significant for the 108 vs. 208 ms comparison (t (9) = 2.41, p<.05).

Table 1 shows the RTs. Overall performance did not differ between the groups (F (1, 18)<1, ns.). However, the effect of SOA, and the interaction between SOA and group were significant (SOA: F (3.4, 60.9) = 20.30, p<.001; SOA×Group: F (3.4, 60.9) = 2.81, p<.05). RTs improved rapidly at early SOAs (8 vs. 108 ms: t (9) = 4.61, p<.01; t (9) = 5.24, p<.01) for both the Distractors absent and the Distractors present group, respectively. At longer SOAs however, RTs remained constant for the Distractors absent group (SOAs 108 vs. 408 ms: t (9)<1, ns.) whereas in the Distractors present group RTs slowed down again when SOA was increased from 108 to 408 ms (t (9) = 4.29, p<.01). Thus, the pattern of RTs further confirmed that of the accuracy scores.

The results of Experiment 2 corroborate the pattern found in Experiment 1. Cue enhanced performance up to about 108 ms, after which the pattern was different for the two groups. Participants who saw targets with distractors, showed a decline at later SOAs, whereas participants receiving only targets had a sustained pattern of attention. Thus it seems that the time course of attentional selectivity is not dependent on differential strategies.

Besides the overall convergence of the results in Experiments 1 and 2, a difference in initial performance was found. Namely, the order of the distractor conditions at 8 ms SOA was reversed in Experiment 2 compared to Experiment 1. Whereas in Experiments 1a and 1b accuracy was initially equal or higher in the distractors absent condition, Experiment 2 showed an opposite pattern. Possible reasons for this could be the between-subjects manipulation used in Experiment 2, or individual differences between participants of different experiments. Most importantly however, irrespective of the small individual variation, the general pattern of results in Experiment 2 coincided with that of Experiment 1 and give further support to the idea that the selectivity aspect of attention is reflected in a transient mechanism.

## Experiment 3

So far we have assumed that the cue recruits attention towards the target, enhances its representation, and selectively shields it against competing objects. This type of spatially selective effect would be consistent with accounts such as the selective tuning model of attention, which assumes facilitation that is specific in the attended location, surrounded by an area of suppression [20], [32]. However, the cue could also function as a general warning signal, leading to an overall improvement in performance with time. Why such an overall enhancement would then be transient in one condition but not in another remains an open question. Nevertheless, to show that we are dealing with selective attention here, we thought it prudent to demonstrate that the cue has a local, selective enhancement effect, by comparing it to a global, non-specific cue. This global cue was the same red box as the selective cue in the previous experiments, but now drawn around the entire display (including distractors) rather than just the target. The target still always appeared at the same central location, hence no spatial shifts were required. Importantly, both the selective cue and the non-selective cue provided the same temporal information about the upcoming target and should thus generate the same overall arousal or alertness.

### 3.1. Methods

Twelve new students (four males, aged 18–27 years, mean 22 years) participated in Experiment 3. We replaced one participant whose overall performance fell below chance level. Stimuli and procedure were the same as in the distractors present condition of Experiment 1b, except for the following. Cuing condition (Local/Global) was varied between blocks. In the Local cue condition a cue surrounded the central target location, identical to the previous experiments. In the Global cue condition, the cue was replaced with a similar red square that extended to surround the whole 6.2°×6.2° area filled with distractors and the central target. SOA was varied in five steps (8, 58, 108, 208, and 408 ms) in both conditions. The order of the blocks was counterbalanced across participants. Both conditions were practiced separately in the beginning of the experiment. Target duration was set to 42 ms. Due to a small technical problem, the target duration for one participant varied between 33 ms in the beginning of the experiment and 42 ms later on. Since this particular participant received more Local cue blocks in the beginning of the experiment, this meant that the average target duration for this participant differed between the conditions, being 38 ms for the Local cue condition and 40 ms for the Global cue condition on average. As the results show, this difference cannot explain the findings (performance was overall worse in the Global cue condition).

### 3.2. Results and Discussion

**Figure 4:**
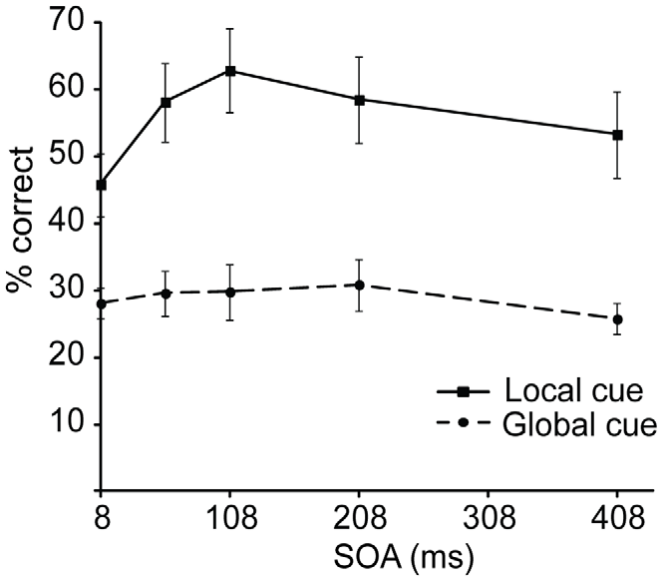
Target identification accuracy for Experiment 3, plotted as a function of SOA and cuing condition.

See Figure 4 for accuracy scores of Experiment 3. Of all trials, 2.1% were excluded because of too fast (<200 ms) or too slow (>5000 ms) responses. Overall accuracy was significantly affected by the cuing condition (F (1, 11) = 25.68, p<.001), with accuracy for the Local cue condition (*M* = .56, *SD* = .21) being better than for the Global cue condition (*M* = .29, *SD* = .11). Also the effect of SOA and the interaction between the cuing condition and SOA were significant (F (4, 44) = 7.9, p<.001; F (4, 44) = 4.48, p<.01, respectively). In the Local cue condition a pattern similar to the previous experiments was observed. Accuracy was first improved when the SOA was increased (8 vs. 108 ms SOA: t (11) = 5.02, p<.001), after which it declined at later SOAs (108 vs. 408 ms SOA: t (11) = 2.47, p<.05). In the Global cue condition there was no difference in accuracy between 8 and 108 ms SOAs (t (11)<1, ns.) nor between 108 and 408 ms SOAs (t (11) = 1.32, ns.).

RTs, shown in Table 1, were marginally affected by the cuing condition (F (1, 11) = 3.6, p = .08), whereas the effect of the SOA was significant (F (4, 44) = 5.88 p<.01). Also the interaction approached significance (F (4, 44) = 2.18, p = .09), suggesting largely the same pattern for RTs as for accuracies. The RTs tended to be overall faster in the Local cue condition, with again a temporary improvement at 108 ms SOA, not seen in the Global cue condition. In the Global cue condition there was only a late rise of RTs, similar to the Local cue condition.

The results of Experiment 3 suggest that for the transient enhancement pattern of attentional selection to occur, a cue at the target location is needed. The global cue did not help in discriminating the target, even though it signalled the appearance of the target display at the same temporal intervals as the local cue. This is consistent with the studies that have measured performance accuracy after a presentation of a non-specific warning signal, as they have either found effects on accuracy at considerably longer SOAs than here [31], [33] or failed to find any modulations of accuracy [21]. In fact, warning signals tend to exert their effects mostly on response preparation [34], which explains the effects on RTs here, while at the same time there was little effect on perceptual accuracy. Furthermore, in warning signal experiments, RTs tend to rise again with the later SOAs [beyond 500 ms; e.g. 10], as was also found here.

Thus, the data indicate that the local cue selectively draws attention to the target, and temporarily protects it from the competing distractors. In contrast, the global signal may have diffused attention across the stimuli, even though the target position was always central and thus known by the participants. In any case, the global signal did not result in the transient pattern of enhancement shown by the local cue. Instead, this transient component appears specific to selective attention, as it is tied to a selective cue, and is only expressed under circumstances of competition.

## Experiment 4

We have argued that the transient time course of attention relates to the presence of competition, in the form of distractors. Alternatively, the accuracy drop for distractors present condition could be associated with a generally higher difficulty level, and not be specific for the selection process. A more difficult task may be more sensitive to subtle changes in attention. Conversely, peak performance in the easier distractors absent condition may have been compressed against ceiling, thus camouflaging a potential drop at later SOAs – although note that performance occasionally rose even further at later SOAs. In the previous experiments we tried to equate for overall difficulty by varying target duration, but we only partially succeeded. Experiment 4 was conducted to explicitly test whether increasing the task difficulty in a single target condition would lead to a similar time pattern as was observed with distractors. We reasoned that if the decline of performance at the long SOAs would be merely dependent on the overall difficulty of the target discrimination task and not on the presence of competing distractors (i.e. the need for selection), the drop should be observable also for some other difficulty manipulation than distractor competition. In Experiment 4, the target duration was therefore reduced to 16 ms to make single target identification more difficult than in any of the previous conditions.

### 4.1. Methods

Ten new students (six males, aged 17–24 years, mean 21 years) participated in Experiment 4. Stimuli and procedure were the same as before, except for the following: Distractors were always absent but instead, the target duration was varied between Short (16 ms) and Long (33 ms). The number of SOA levels was reduced to four: 58, 108, 208, and 408 ms.

### 4.2. Results and Discussion

**Figure 5:**
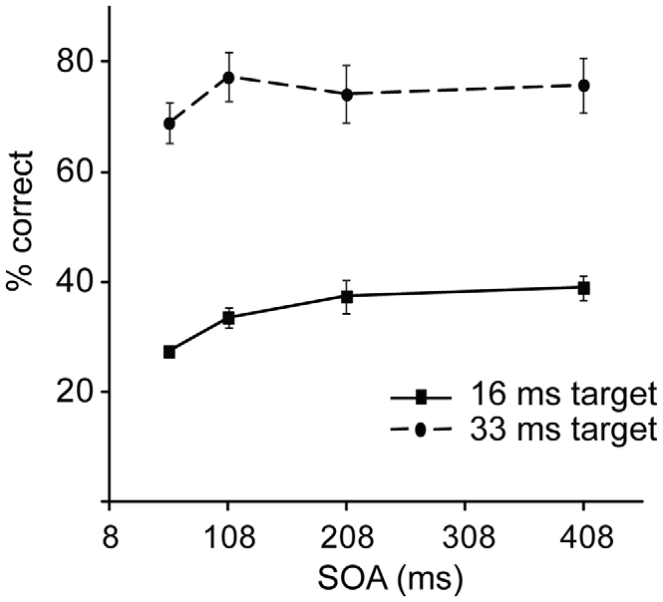
Target identification accuracy for Experiment 4, plotted as a function of SOA and target duration.

Figure 5 shows the accuracy scores for Experiment 4. Due to too slow (>5000 ms) or fast (<200 ms) RTs, 1.5% of all trials was excluded. An ANOVA with Target duration (Short vs. Long) and SOA (58, 108, 208, and 408 ms) revealed a significant overall effect of Target duration on accuracy (F (1, 9) = 141.87, p<.001) as the proportion correct for Short targets was less than for Long targets (*M* = .34, *SD* = .08, being however above chance: t (9) = 5.57, p<.001, vs. *M* = .74, *SD* = .14, respectively). Also the main effect of SOA and the interaction between Target duration and SOA were significant (F (3, 27) = 11.40, p<.001; F (3, 27) = 3.71, p<.05; respectively). Accuracy improved in both Target duration conditions when SOA was increased from 58 to 108 ms (Short targets: t (9) = 3.31, p<.01; Long targets: t (9) = 4.23, p<.01). At longer SOAs accuracy improved even further for Short targets but remained constant for Long targets (108 vs. 408 ms: t (9) = 3.60, p<.01; t (9)<1, ns., respectively). There were no significant effects on RTs (Target duration: F (1, 9) = 1.48, ns.; SOA: F (1.3, 12) = 2.73, ns.; interaction: F (1.5, 13.5) = 0.80, ns.).

Experiment 4 suggests that merely increasing task difficulty (by reducing target duration) does not result in the transient time pattern that was found when distractors were present in the target display. More concretely, accuracies at longer SOAs did not decline when the target duration was shortened. If anything, results of Experiment 4 suggest that the task performance improved at longer SOAs for the short duration targets, as compared to the long duration targets. Thus, the more difficult the task, the more observers may utilize the longer cue-target SOAs. In contrast, the transient performance pattern appears to specifically reflect attentional selection processes operating when a target competes for representation against other proximal stimuli.

## Discussion

The objective of this study was to investigate the time course of the selection component of attention, in the absence of spatial shifting. Four experiments showed an initial rapid enhancement of performance when target was cued at central fixation, up to about 100 ms. Importantly, we showed that at late SOAs the pattern of performance was dependent on the presence of distractors. Performance declined after the peak when distractors were surrounding the target. In contrast, when the target was presented alone, performance remained constant or even gradually improved further with increasing SOA. This pattern did not depend on potential overall strategies, as it was observed with a (mixed) within-subject design (Experiments 1 and 1b), as well as with a (blocked) between-subjects design (Experiment 2). It also occurred irrespective of whether target duration was varied to compensate for difficulty (Experiments 1 and 2) or kept constant (Experiment 1b). Experiment 1 showed that the cue resulted in an enhancement of performance relative to when no cue was present. Experiment 3 showed that the observed enhancement required a selective signal at the target location, and was not evoked by the sole temporal information provided by a global, non-specific cue. Finally, Experiment 4 showed that merely increasing the overall task difficulty, by shortening the target duration even further, did not result in a decrease of performance at late SOAs in the target only condition, suggesting that distractors are necessary for the drop in performance to occur. We take these results as evidence for a transient time course of attention, but specifically the selection component of it.

### The transience of selection

The transient pattern observed in the distractors present condition strongly resembles the one that has been reported earlier in spatial orienting studies [2], [5], [6], [7]. The current findings show that the selective attention has a transient pattern, also when all relevant items are consistently presented at fixation, and there is no need for spatial shifting. In other words, the activation of the re-orienting component is not necessary for attention to be transient. However, whether it is sufficient, cannot be answered based on the present results. An interesting question for future research is whether the selectivity accounts for the transient attentional effects also when the task requires spatial re-orienting. In addition, the results confirm that the transient pattern of attention occurs at central vision. This is important because attention has been suggested to have differential effects on foveal and peripheral vision [35], [36], [but see 37], and transient attention has often been linked specifically to peripheral vision [though this have never been previously tested]; [ 38], [39], [40], [41,42].

Most importantly though, the present study suggests that the transient character of performance enhancement belongs to the selective aspect of attention, as it only occurred, or was only expressed, when distractors were present. The effect of distractors in spatial cuing has been studied previously [6]. Müller and Findlay presented their participants with targets alone, or together with three distractor items. Both the single and multiple item condition resulted in a rapid enhancement, followed by decay in performance. These results appear contradictory with what we found, namely that the transient pattern occurred only when the target was presented among competing items. This discrepancy between these two studies might be spurious however. As noted by Müller and Findlay themselves, the four possible target locations in their study were rapidly replaced by individual masks in both single item and multiple item conditions after the brief presentation of the target. Moreover, in both conditions they used box-shaped place holders at all possible target locations. In other words, even the single item condition contained multiple items. Assuming that both conditions in the study of Müller and Findlay contained competing elements, their results seem to be consistent with the present conclusion: The attentional selection, applied to a target among competing objects, has a transient time course.

The finding that the time course of performance depends on the presence of competing distractors is consistent with theories that stress the role of competition in selective attention [18], [19]. These theories suggest that attention has an effect only, or especially, when one or more non-target items are competing with the target for representation, and a representational ambiguity needs to be resolved. Attention is then expressed as a bias of this competition in favour of salient, cued or otherwise behaviourally relevant objects, while irrelevant objects are suppressed. Furthermore, this suppression may be closely tied to the surroundings of the attentional focus, as suggested by the selective tuning model [20]. What our data suggest is that this biasing of competition, resolution of ambiguity, or selective tuning is only temporary: After a few hundred milliseconds the cue starts to lose its selective ability to protect the target against distractors.

An important question that remains is why attention, when facing competition, behaves in a transient fashion. One possibility is that the transience reflects mere habituation of the attentional response, such that after an initial strong burst of activity, neuronal fatigue causes the selected location to be less resistant to competing objects. Another possibility is that the transience reflects an automatic disengagement process, and as such has a clear functional purpose. When faced with a relevant or salient object, selective attention may actively lock on to it for about 100 to 200 ms – a time period that is usually sufficient for identification of even the most complex stimuli [43] – before starting to move away or broaden its focus again. It may then take another while before it is fully disengaged, resulting in an estimated dwell time of around 250 ms or more [44], [45]. When there is only a single object in the field, there may be no signal to disengage, or the disengagement is not so detrimental since there are no competing stimuli.

In this respect the mechanism underlying the attentional decline bears resemblance to that of IOR, which is thought to reflect a mechanism that inhibits attended locations, in order to prevent attention from returning to them [46], [47]. This resemblance is strengthened by the fact that transient attention pattern and IOR have both been previously observed in spatial cuing tasks, and the performance decline in both paradigms overlaps in time, occurring beyond SOAs of approximately 200 ms. However, there also appear to be differences. Whereas IOR is measured when observers are required to reorient attention between multiple locations, and is especially apparent when observers need to make (or suppress) an eye movement towards a peripheral location, here we measured a performance decline even when the cue and target were always presented at the same, central, location. This means at least that the presumed disengagement mechanism can be measured without the need for attention to move away (and move back again). Furthermore, we found a decline only when distractors were present, whereas IOR typically also occurs for presentations of a single cued target, without distractors. Future studies should compare transient attentional enhancement and IOR more directly in order to investigate to what extent they reflect the same mechanism.

### Masking

May masking account for the transient performance pattern that we found here? Could the cue be masking the target, and thus cause the transient pattern? Paracontrast (forward) masking has been found to have a non-monotonic effect on perception, with brief suppression at very short (10–30 ms) mask-target intervals, some relief at around 40 ms, followed by a longer lasting suppression up to about 450 ms SOA [48]. According to Breitmeyer and colleagues, the non-monotonic pattern occurs because the mask triggers an additional “transient enhancement” mechanism, which “gates” the subsequent target input. In fact, such a transient gating function is not unlike the attentional function that we propose here. What we show is that it must be a local enhancement, and one that temporarily protects against competition. In any case, although we cannot fully exclude masking, we believe its contribution is at most minor. Note that in the present study the cue seemed to cause nothing but enhancement, and that this enhancement was sustained even under difficult conditions of very brief target presentation (Experiment 4). Such briefly presented targets should have been very sensitive to a forward mask. Instead, the transient character appeared to depend on the presence of distractors. It is possible that the late decline in the distractors present condition may have been enhanced by crowding, as suggested by a recent study of Vickery, Shim, Chakravarthi, Jiang, and Luedeman [49]. They found that target identification was impaired by a surrounding square (similar to our cue), and that this effect was increased by target flanking distractors. Although Vickery et al. only tested concurrent presentations, it may be the case that this enhanced crowding effect also holds for long SOAs (but not intermediate ones). It is questionable whether crowding is very strong for the foveal stimuli and inter-item spacing that we used [16]. More importantly though, note again that we found performance to be overall enhanced by the cue, not suppressed. In other words, if anything, the cue here appears to induce a mechanism that helps to temporarily overcome the detrimental effects of crowding, in line with a transient attention component biasing the competition between multiple elements.

### Relation to other transient attentional effects

We will further discuss two related phenomena that have been assumed to reflect a transient enhancement of attention at central presentation. First, the recently found *attentional boost effect* shows a temporary processing enhancement of centrally presented objects [50], [51]. The attentional boost effect has been observed as a peak in recognition memory for pictures that coincide with a target item of a second, unrelated task, both presented in a central stream, typically at about two items per second. The exact mechanism behind attentional boost effect is still unknown, but it has been suggested to stem from the phasic activation of locus coeruleus [50], [51], which might cause a general processing enhancement across the visual field. Consistent with a more general effect, it has been shown that the attentional boost effect occurs also when pictures are combined with auditory targets [50]. According to Swallow and Jiang, the attentional boost effect is separable from more selective attentional cuing because of their differential time course. No attentional boost effect was found when a cuing target preceded a picture by 100 ms, as opposed to presenting the two concurrently [51]. This is in strong contrast with the present results, which show the largest cuing benefits at 100 ms SOA, and the smallest at concurrent presentation. In addition, we found the transient attentional pattern only for a local signal, and only when distractors were present, suggesting a rather focused and selective attentional effect. Hence we agree that the attentional boost effect and the present cuing effects are likely to be different.

Second, a number of temporal attention theories [26], [27], [52], [53], [54] suggest that transient attentional enhancement underlies the attentional blink, a temporal impairment in identifying the second of two targets presented in RSVP [27], [55]. According to these theories the attentional blink either occurs because attention transiently enhances the post-target distractor processing, which results in subsequent target inhibition, or because transient attentional enhancement is blocked by the first target processing, and is thus not available for the second target. Common to these theories is that they assume the first target to initiate a transient attentional enhancement response, identical to which has been found earlier in peripheral cuing. As pointed out in the introduction, this assumption has been however complicated by the fact that in RSVP all items are typically presented at the same location, whereas in peripheral cuing spatial shifts are required. Here we present direct evidence for a transient pattern of selective attention in invariable, central presentations, without the conundrums of RSVP.

### Conclusions

It is shown that the time course of the selection component of attention is transient. Cuing at central location enhances performance rapidly irrespective of whether distractors surround the target or not. At the longer SOAs however performance is dependent on competition: performance is sustained if targets are presented alone, but in presence of distractor objects, performance is transient, as it declines with time. Altogether, the present study provides evidence for a common transient time course of selection that has been assumed in studies of both spatial and temporal attention.

## References

[1] Posner MI (1980). Orienting of attention.. Quarterly Journal of Experimental Psychology.

[2] Nakayama K, Mackeben M (1989). Sustained and transient components of focal visual attention.. Vision Research.

[3] Chastain G, Cheal ML (1998). Automatic versus directed attention with single-element and multiple-element precues.. Visual Cognition.

[4] Kristjánsson Á, Mackeben M, Nakayama K (2001). Rapid, object-based learning in the deployment of transient attention.. Perception.

[5] Mackeben M, Nakayama K (1993). Express attentional shifts.. Vision Research.

[6] Müller HJ, Findlay JM (1988). The effect of visual attention on peripheral discrimination thresholds in single and multiple element displays.. Acta Psychologica.

[7] Müller HJ, Rabbitt PMA (1989). Reflexive and voluntary orienting of visual attention: time course of activation and resistance to interruption.. Journal of Experimental Psychology: Human Perception and Performance.

[8] Scharlau I, Ansorge U, Horstmann G (2006). Latency facilitation in temporal-order judgments:Time course of facilitation as a function of judgment type.. Acta Psychologica.

[9] Fan J, McCandliss BD, Sommer T, Raz A, Posner MI (2002). Testing the efficiency and independence of attentional networks.. Journal of Cognitive Neuroscience.

[10] Posner MI, Boies SJ (1971). Components of attention.. Psychological Review.

[11] Posner MI, Petersen SE (1990). The attention system of the human brain.. Annual Review of Neuroscience.

[12] Raz A, Buhle J (2006). Typologies of attentional networks.. Nature Reviews Neuroscience.

[13] Posner MI, Walker JA, Friedrich F, Rafal RD (1984). Effects of parietal injury on covert orienting of attention.. Journal of Neuroscience.

[14] Posner MI, Walker JA, Friedrich F, Rafal RD (1987). How do the parietal lobes direct covert attention?. Neuropsychologia.

[15] Bouma H (1970). Interaction effects in parafoveal letter recognition.. Nature.

[16] Levi DM (2008). Crowding- an essential bottleneck for object recognition: a mini-review.. Vision Research.

[17] Kahneman D, Treisman A, Burkell J (1983). The cost of visual filtering.. Journal of Experimental Psychology: Human Perception and Performance.

[18] Desimone R, Duncan J (1995). Neural mechanisms of selective visual attention.. Annual Review of Neuroscience.

[19] Luck SJ, Girelli M, McDermott MT, Ford MA (1997). Bridging the gap between monkey neurophysiology and human perception: an ambiguity resolution theory of visual selective attention.. Cognitive Psychology.

[20] Tsotsos JK, Culhane SM, Wai WYK, Lai Y, Davis N (1995). Modeling visual attention via selective tuning.. Artificial Intelligence.

[21] Los SA, Schut MLJ (2008). The effective time course of preparation.. Cognitive Psychology.

[22] Müller-Gethmann H, Ulrich R, Rinkenauer G (2003). Locus of the effect of temporal preparation: Evidence from the lateralized readiness potential.. Psychophysiology.

[23] Niemi P, Näätänen R (1981). Foreperiod and simple reaction time.. Psychological Bulletin.

[24] Weichselgartner E, Sperling G (1987). Dynamics of automatic and controlled visual attention.. Science.

[25] Dux PE, Marois R (2009). The attentional blink: a review of data and theory.. Attention, Perception, & Psychophysics.

[26] Olivers CNL, Meeter M (2008). A Boost and bounce theory of temporal attention.. Psychological Review.

[27] Raymond JE, Shapiro KL, Arnell KM (1992). Temporary Suppression of Visual Processing in an RSVP Task: An Attentional Blink?. Journal of Experimental Psychology: Human Perception and Performance.

[28] Kristjánsson Á, Sigurdardottir HM (2008). On the benefits of transient attention across the visual field.. Perception.

[29] Shiu L, Pashler H (1995). Spatial attention and vernier acuity.. Vision Research.

[30] Hackley SA, Valle-Inclán F (1998). Automatic alerting does not speed late motoric processes in a reaction-time task.. Nature.

[31] Rolke B, Hofmann P (2007). Temporal uncertainty degrades perceptual processing.. Psychonomic Bulletin & Review.

[32] Cutzu F, Tsotsos JK (2003). The selective tuning model of attention: psychophysical evidence for a suppressive annulus around an attended item.. Vision Research.

[33] Rolke B (2008). Temporal preparation facilitates perceptual identification of letters.. Perception & Psychophysics.

[34] Hackley SA, Valle-Inclán F (2003). Which stages of processing are speeded by a warning signal?. Biological Psychology.

[35] Handy TC, Khoe W (2005). Attention and sensory gain control: a peripheral visual process?. Journal of Cognitive Neuroscience.

[36] Yeshurun Y, Carrasco M (1998). Attention improves or impairs visual performance by enhancing spatial resolution.. Nature.

[37] Frey HP, Kelly SP, Lalor EC, Foxe JJ (2010). Early spatial attentional modulation of inputs to the fovea.. Journal of Neuroscience.

[38] Carrasco M, Penpeci-Talgar C, Eckstein M (2000). Spatial covert attention increases contrast sensitivity across the CSF: support for signal enhancement.. Vision Research.

[39] Carrasco M, Talgar CP, Cameron EL (2001). Characterizing visual performance fields: effects of transient covert attention, spatial frequency, eccentricity, task and set size.. Spatial Vision.

[40] Kristjánsson Á, Nakayama K (2003). A primitive memory system for the deployment of transient attention.. Perception & Psychophysics.

[41] Ling S, Carrasco M (2006). Sustained and transient covert attention enhance the signal via different contrast response functions.. Vision Research.

[42] Liu T, Pestilli F, Carrasco M (2005). Transient attention enhances perceptual performance and fMRI response in human visual cortex.. Neuron.

[43] Potter MC (1976). Short-term conceptual memory for pictures.. Journal of Experimental Psychology: Human Learning and Memory.

[44] Theeuwes J, Godijn R, Pratt J (2004). A new estimation of the duration of attentional dwell time.. Psychonomic Bulletin & Review.

[45] Ward R, Shapiro K (1999). Visual attention is no faster than the eyes.. The limits of attention: Temporal constraints on human information processing.

[46] Klein RM (2000). Inhibition of return.. Trends in Cognitive Sciences.

[47] Posner MI, Cohen Y, Bouma H, Bouwhuis DG (1984). Components of visual orienting.. Attention and Performance X: Control of Language Processes.

[48] Breitmeyer BG, Kafaligönül H, Öğmen H, Mardon L, Todd S (2006). Meta- and paracontrast reveal differences between contour- and brightness-processing mechanisms.. Vision Research.

[49] Vickery TJ, Shim WM, Chakravarthi R, Jiang YV, Luedeman R (2009). Supercrowding: Weakly masking a target expands the range of crowding.. Journal of Vision.

[50] Swallow KM, Jiang YV (2010). The attentional boost effect: transient increases in attention to one task enhance performance in a second task.. Cognition.

[51] Swallow KM, Jiang YV (2011). The role of timing in the attentional boost effect.. Attention, Perception, & Psychophysics.

[52] Bowman H, Wyble B (2007). The simultaneous type, serial token model of temporal attention and working memory.. Psychological Review.

[53] Nieuwenhuis S, Gilzenrat MS, Holmes BD, Cohen JD (2005). The role of the locus coeruleus in mediating the attentional blink: a neurocomputational theory.. Journal of Experimental Psychology: General.

[54] Wyble B, Bowman H, Potter MC (2009). Categorically defined targets trigger spatiotemporal visual attention.. Journal of Experimental Psychology: Human Perception and Performance.

[55] Broadbent DE, Broadbent MHP (1987). From detection to identification: response to multiple targets in rapid serial visual presentation.. Perception and Psychophysics.

